# Compact and Fully Integrated LED Quantum Sensor Based on NV Centers in Diamond

**DOI:** 10.3390/s24030743

**Published:** 2024-01-24

**Authors:** Jens Pogorzelski, Ludwig Horsthemke, Jonas Homrighausen, Dennis Stiegekötter, Markus Gregor, Peter Glösekötter

**Affiliations:** 1 Department of Electrical Engineering and Computer Science, Münster University of Applied Sciences, Stegerwaldstr. 39, D-48565 Steinfurt, Germany; 2 Department of Engineering Physics, Münster University of Applied Sciences, Stegerwaldstr. 39, D-48565 Steinfurt, Germanymarkus.gregor@fh-muenster.de (M.G.)

**Keywords:** NV center, diamond microcrystal, magnetometry, integrated quantum sensor

## Abstract

Quantum magnetometry based on optically detected magnetic resonance (ODMR) of nitrogen vacancy centers in diamond nano or microcrystals is a promising technology for sensitive, integrated magnetic-field sensors. Currently, this technology is still cost-intensive and mainly found in research. Here we propose one of the smallest fully integrated quantum sensors to date based on nitrogen vacancy (NV) centers in diamond microcrystals. It is an extremely cost-effective device that integrates a pump light source, photodiode, microwave antenna, filtering and fluorescence detection. Thus, the sensor offers an all-electric interface without the need to adjust or connect optical components. A sensitivity of 28.32$\;\mathrm{nT}/\sqrt{\mathrm{Hz}}$ and a theoretical shot noise limited sensitivity of 2.87 $\mathrm{nT}/\sqrt{\mathrm{Hz}}$ is reached. Since only generally available parts were used, the sensor can be easily produced in a small series. The form factor of (6.9 × 3.9 × 15.9) ${\mathrm{mm}}^{3}$ combined with the integration level is the smallest fully integrated NV-based sensor proposed so far. With a power consumption of around 0.1W, this sensor becomes interesting for a wide range of stationary and handheld systems. This development paves the way for the wide usage of quantum magnetometers in non-laboratory environments and technical applications.

## 1. Introduction

In recent years, negatively charged NV centers in diamond have become established in the field of quantum-based sensing. NV centers can be used to build highly sensitive magnetic field sensors even in the $\mathrm{fT}/\sqrt{\mathrm{Hz}}$ range [1,2,3]. These can be kept extremely small with spatial resolutions down to atomic size [4,5,6,7]. This sensor technology can also measure magnetic fields very accurately combined with low energy and space requirements [8]. NV centers can also be used to measure temperatures [9,10,11,12], electric fields [13] and there are also applications in the field of quantum computing [14,15]. Other magnetic sensing protocols using the NV center include an all-optical approach using spin mixing in the NV ground state [16,17,18] or measurement of the infrared absorption of the infrared transition with near shot-noise limited sensitivity [19]. As they are a solid-state system in diamond, the sensors can be operated at room temperature. Therefore the structure can be kept less complex, as cryogenic temperatures are not required.

**Figure 1:**
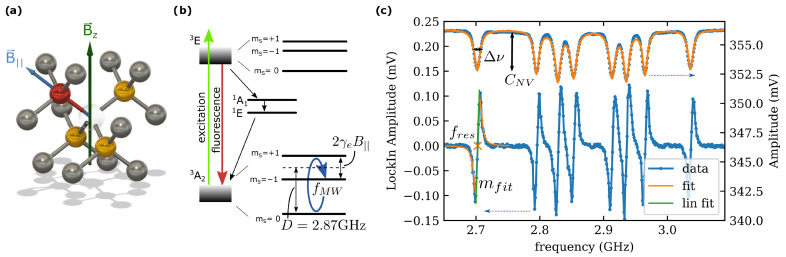
(**a**) Diamond crystal structure formed by carbon atoms (grey) with nitrogen atom (red) and adjacent vacancy forming a nitrogen vacancy (NV) center. NV centers are formed in all axes of the diamond lattice (indicated by yellow-colored carbon atoms). Green arrow indicates an external magnetic field $B_{z}$ whereas the blue arrow indicates the vectorial projection on one of the NV-axis $B_{\left|\right|}$ (**b**) simplified energy diagram of the NV center. (**c**) Example spectrum measured by multimeter (upper curve—related to right axis) and lock-in amplifier (lower curve—related to left axis). Contrast of the resonance $C_{NV}$ and full width at half maximum Δν are extracted from fit function. The slope of the resonance is extracted from a fit to the demodulated signal of the LIA.

The NV center is a point defect in diamond. The diamond crystal structure is shown in Figure 1a. Two of the carbon atoms are replaced by a nitrogen atom (red) and an adjacent vacancy. For an ensemble of NV centers in a solid diamond, all four orientations within the tetrahedral structure of the diamond are possible (indicated by yellow atoms).

A negatively charged NV center is a spin S=1 system with spin triplets in the ground state ground state (A23) and in the excited state (E3) (cf. Figure 1b) The optical excitation of the ground state is spin-conserving. The decay of electrons in the $m_{s}=0$ spin state leads to fluorescence with a dominant wavelength of 637nm, while the $m_{s}=\pm 1$ state has a higher probability of non-radiative transitions to the A11 singlet state. Manipulation of the electron spin state with microwave magnetic field resonant to the electron spin transitions in the ground state A23 will therefore decrease the fluorescence emitted by the NV center.

The magnetic sensing capability of the NV center is given by the interaction of an external magnetic field $B_{z}$ (green arrow—cf. Figure 1a) with the electron spin. Due to the Zeeman effect, the $m_{s}=\pm 1$ electron spin states are shifted by the projected parallel component $B_{\left|\right|}$ (blue arrow—cf. Figure 1a). This shift can be read out in optically detected magnetic resonance (ODMR) measurements. Without any applied magnetic field, a zero field splitting (ZFS) is still visible due to internal crystal strain. The ZFS center frequency (D=2.87GHz at room temperature) shifts with temperature and is used for temperature sensing [9,20].

For NV ensembles, in which NV centers are aligned along four crystal axes, a sweep of the microwave frequency while observing the fluorescence intensity will yield a total of eight dips in fluorescence, corresponding to the $m_{s}=+1$ and $m_{s}=-1$ levels for each of the four NV quantization axes (cf. Figure 1c).

When examining NV-based sensors a distinction can be made between fiber-based sensors, partial fiber-based sensors and fully integrated sensors. Fiber-based sensors have the pump light source and detection outside the sensor head. Partial fiber-based sensors integrate the pump light source or at least the detection in the sensor head. Fully integrated sensors integrate all optical components and provide an all-electric interface.

The advantage of fiber-based systems is that the sensor head can be made very small [21,22,23]. The laser systems are not limited in their size and performance. Fiber-based systems have the benefit that the light sources which generate passive heat are not installed directly in the sensor head and are therefore not restricted in their size and performance. This makes them ideal for biological applications as they offer more degrees of freedom. In addition, the overall size of the sensor head can be reduced to micrometer diameters [21], which is currently not possible due to the component sizes in integrated sensors. However, the combination of sensitivity and form factor of these sensors have mostly been achieved under laboratory conditions with the aid of additional free beam optics. These optical components are often susceptible to mechanical stress, vibrations and for multimode fibres also fiber bending. Also, optical setups cause high costs and often high additional form factors.

Partial fiber-based sensors integrate the photodiode (PD) and continue to use an external laser [1,24,25,26]. An outstanding form factor with a high integration level in this category is reached by Kim et al. [27] but is currently limited in the reached sensitivity of 32$\;\mu T/\sqrt{\mathrm{Hz}}$. Attempts are also being made to additionally integrate laser diodes as pump light sources inside the sensor head [28,29,30,31] and thus to be fully integrated. However, these devices are relatively large (above $1\;{\mathrm{cm}}^{3}$), which is partly due to the available size of the laser diodes.

For fully integrated sensors, other pump light sources such as light-emitting diodes (LED) could be a possibility. However, fully integrated LED-based sensors have only been marginally investigated so far [11,32]. The aim of this work is to show that fully integrated sensors enable significantly simpler setups compared to partially integrated sensors or fiber-based systems. This could pave the way for specific industrial applications, especially as they provide an all-electric interface. The aim is not necessarily to compete with the highest sensitivities, but rather to achieve a simple and cost-effective design that still offers good performance. By using an LED, it is possible to reduce both the power consumption compared to a laser and the complexity of the control system. For fully integrated sensors LEDs offer a smaller form factor than laser diodes or similar and are therefore useful as they can reduce the size of the entire sensor head. By using surface mount devices (SMD) and an extremely reduced and modular design, we present a fully integrated LED-based NV magnetometer that reduces the size of the so far smallest fully integrated sensor by Stürner et al. [32] by factor 7 to $0.42\;{\mathrm{cm}}^{3}$. Also, the sensitivity is slightly improved to 28.32$\;\mathrm{nT}/\sqrt{\mathrm{Hz}}$. Major progress in the area of cost savings is achieved by using diamond microcrystals.

## 2. Materials and Methods

To build an NV center-based sensor, several key technology components have to be combined: excitation light source, diamond microcrystal, microwave (MW) antenna, red fluorescence filter and photodiode for detection. In a fully integrated sensor, all optical components are included and the sensor has only an electric interface which can be characterized. Difficulties in integration are the interaction between the different signals, heating of the components as well as mechanical stability. The fundamental mechanical sensor structure is described first, followed by a description of the components used.

### 2.1. Sensor Structure and Mounting

**Figure 2:**
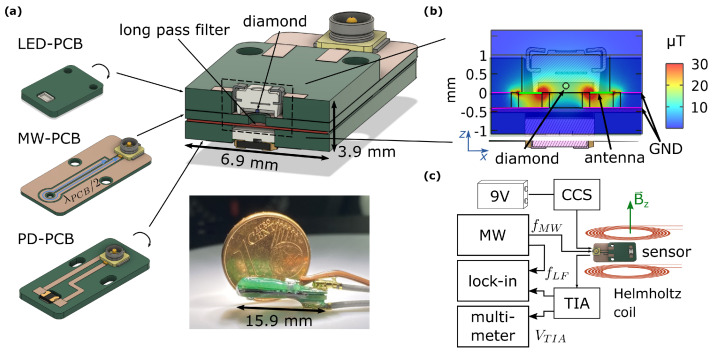
(**a**) Sensor setup containing LED-PCB, microwave (MW) antenna structure (MW-PCB) and the PCB to mount the photodiode (PD-PCB), as well as the 100μm thick filterfoil between MW-PCB and PD-PCB. The overall size is $\left(6.9\times 3.65\times 15.9\right)\;{\mathrm{mm}}^{3}$. (**b**) Simulation of the field distribution inside the sensor at 10dBm microwave power. (**c**) Electronic block diagram. A 9V battery feeds a lab-built constant current source for 30mA LED current. The microwave source generates a frequency-modulated microwave whose LF frequency is used as the demodulation frequency for the lock-in amplifier (LIA). The photocurrent is fed to a lab-built TIA which provides input voltage for the LIA and a multimeter.

The sensor is designed in a stacked construction as shown in Figure 2a. The main components are three printed circuit boards (PCBs). These can easily be manufactured since standard design rules were used. The first PCB (LED-PCB) has contacts to solder the LED and connect the LED feed line. A 150μm sized diamond microcrystal (MDNV150umHi30mg, Adámas Nanotechnologies, Raleigh, NC, USA) is then fixed over the light-emitting chip with an optical adhesive (NOA61, Norland Products, Jamesburg, NJ, USA), onto the epoxy resin that fills the LED housing.

The diamond volume is approximated with a sphere that has a diameter of 170μm as the diamonds are slightly larger than specified by the manufacturer. This results in a diamond volume of approximately $0.02\;{\mathrm{mm}}^{3}$. The concentration of NV centers in the diamonds amounts to 2.5–3 ppm [33] which leads to a strong fluorescence signal that can be detected using standard photodiodes. The second PCB provides the MW field and is fabricated as a λ/2 microwave antenna (MW-PCB). The microwave antenna is wrapped around a hole in the MW-PCB, which enables the collection of the fluorescence signal. The photodiode is inserted into the third PCB (PD-PCB). An optical filter (622 nm Longpass Wratten Colour Filter 75 × 75 × 0.1 mmthk, Knight Optics, Harrietsham, Kent, UK) is placed between MW-PCB and PD-PCB. MW-PCB and PD-PCB are both connected via HIROSE U.FL connectors and coaxial cables. The PCBs can be aligned via removable fitting screws. In the final step, the PCBs are fixed with UV adhesive to achieve mechanical stability.

### 2.2. Microwave Design

The microwave antenna design is based on an omega structure (cf. Figure 2a), whereby the loop of the MW trace leads to a concentration of field strength and an improvement of the field homogeneity around the hole. The length of the antenna is chosen to be exactly half the wavelength of the center frequency of the NV center ground state. This ensures that a minimum of the electric field will be in the center of the resonator length and therefore a maximum of the magnetic field is reached in the hole of the PCB. The resonator length of the PCB trace is calculated as:

(1) $$\lambda_{PCB}/2=\frac{v_{f}\cdot c}{f_{MW}}/2=\frac{c}{\sqrt{\epsilon_{r_{core}}}f_{MW}}/2=24.62\;\mathrm{mm}$$

where $\lambda_{PCB}$ is the wavelength of the electromagnetic wave in a copper trace on a PCB, $v_{f}=1/\sqrt{\epsilon_{r_{core}}}=1/\sqrt{4.5}$ is the velocity factor of the core material with $\epsilon_{r_{core}}=4.5$ given by the PCB manufacturer, *c* is the speed of light and $f_{MW}$ is the designated resonance frequency, here chosen as $f_{MW}=2.87\;\mathrm{GHz}$. The MW trace is wound around the mechanical hole as well as the manufacturing guidelines allow. Also, the antenna is set up as a coplanar waveguide with a ground plane to reduce electromagnetic coupling into the photodiode traces underneath the ground plane. The calculated length is used for setting up a COMSOL Multiphysics simulation of the antenna geometry of the MW-PCB. The simulation shows the effective field guidance (cf. Figure 2b). Almost the entire field that could interfere with the photodiode is guided into the ground plane (GND). In the diamond area, a uniform field distribution over the whole diamond volume is achieved with a maximum deviation from the z-axis of $\theta_{max}=2.83{}^{\circ}$ and an average deviation $\theta_{av}=1.53{}^{\circ}$ where θ is the angle between the magnetic field component in z-direction and the total component of the magnetic field. The calculated values are comparable to widely used geometries of NV center antennas [34,35].

### 2.3. Optical Components

**Figure 3:**
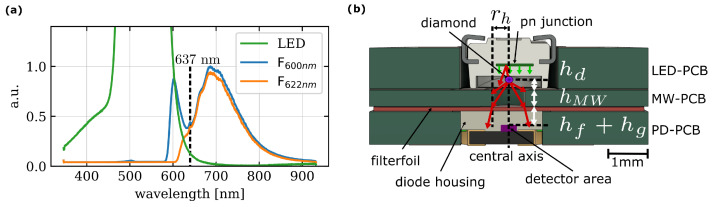
(**a**) Emission spectra for the LED (green) only, fluorescence spectra LED with diamond microcrystal after passing through long pass filter with cut-on wavelength at 600nm (blue) and 622nm (orange). The spectra are recorded by fiber-coupled spectrometer (Ocean HDX, Ocean Insight, Orlando, FL, USA) whereas PD-PCB is replaced by a focusing lens to couple into the fiber. Integration time is set to 1s. (**b**) Model of light paths showing the light path of the fluorescence emitted by the diamond.

An indium gallium nitride LED (150224GS73100, Wuerth Elektronik eiSos GmbH & Co. KG, Waldenburg, Germany) with 525nm dominant wavelength [36] is used as an excitation light source. A directional light output of 1500mcd is given in the datasheet [36]. The LED is inserted through the PCB and then soldered on the top side so that it is fixed on a flat surface with the underside of the LED PCB. The emission spectra of the LED slightly overlap with the fluorescence spectra of the diamond (Figure 3a) which normally does not appear in laser applications. For LED applications, the filter must be selected appropriately. A 600nm filter causes residual light from the LED to worsen the signal-to-noise ratio. Consequently, we use a filterfoil with 622nm cutoff wavelength (622FWP7575, Knight Optics, Harrietsham, Kent, UK). To collect the fluorescence signal a SMD photodiode (VEMD1060X01, Vishay Intertechnology Inc., Malvern, PA, USA) is mounted similarly to the LED through the PCB. The cut-out in the PD-PCB is shifted from the center to place the detector area central underneath the hole of the MW-PCB. This type of mounting allows the alignment and therefore the beam path to be optimized. The holes in the PCB determine the positions of the components. The screws for connecting the PCBs in turn align the PCBs to each other. For the beam path, the assumption is made that the diamond is located about 100μm above the hole and acts as a point emitter (Figure 3b). At a maximum angle of α=43.0°, the rays hit the filter and are refracted there. Because reflections are not expected, the transmission from filter to photodiode is considered consistent. With the filter height $h_{f}=100\;\mu m$ and the thickness of the epoxy between the top of the photodiode to the sensor area $h_{g}=150\;\mu m$, the result is an area where fluorescence illuminates a detector with a radius $r_{fl}$ of

(2) $$r_{fl}=r_{h}+tan(\delta )\cdot \left(h_{f}+h_{g}\right)=r_{h}+\tan\left(\arcsin\left(\frac{sin(\alpha )\cdot n_{air}}{n_{f}}\right)\right)\cdot \left(h_{f}+h_{g}\right)=629.3\;\mu m$$

where $r_{h}$ is the hole radius of the MW PCB, $h_{f}$ is the thickness of the filter, $h_{g}$ is the thickness of the glass above the detector area, $n_{f}$ is the refractive index of the filter and $n_{air}$ is the refractive index of air. This gives enough room to place the PD PCB and to irradiate the entire detector surface.

### 2.4. Measurement Setup

To read out fluorescence signals, a customized transimpedance amplifier (TIA) is used described in a previous publication [37]. The TIA output voltage is fed into a lock-in amplifier (LIA) (MFLI, Zurich Instruments, Zurich, Switzerland). The microwave signal is generated by a vector signal generator (SMBV100B, Rhode & Schwarz, Munich, Germany). The LED is driven by a lab-built constant current source (CCS), powered by a 9V block battery and adjusted to 30mA output current. Furthermore, a multimeter (GDM9061, GW-Instek, Taipeh, Taiwan) is used to measure the output voltage of the TIA or the temperature with a thermocouple type K. A block diagram is shown in Figure 2c.

To provide a bias magnetic field and impose oscillating fields a pair of Helmholtz coils are used and calibrated with a Hall effect sensor (SS94A2D, Honeywell, Charlotte, NC, USA). The current is provided by a four-quadrant power supply (TOE 7621, Toellner Electronic Instrumente GmbH, Herdecke, Germany) set up as a voltage-controlled current source.The input voltage is provided by a signal generator output of an oscilloscope (RTA4004, Rhode & Schwarz, Munich, Germany).

### 2.5. Output Signal Model

The presence of an arbitrary magnetic field is assumed. Due to the Zeeman effect, resonances spilled to up to eight resonances as shown in Figure 1c. A fluorescence resonance dip measured as the output voltage of the TIA $V_{T}$ as a function of the frequency $f_{MW}$ can be approximated with a Lorentzian line shape and, therefore, the whole output signal as the summation of eight Lorentzian dips.

(3) $$V_{T}=V_{0}-\sum_{i=1}^{8}\frac{C_{NV_{i}}}{1+{\left(2\left(f_{MW}-f_{res_{i}}\right)/\Delta \nu_{i}\right)}^{2}}$$

where $V_{0}$ is the measured voltage in the non-resonant case and $f_{res_{i}}$ are the resonance frequencies of the individual dips. $\Delta \nu_{i}$ is the parameter that characterizes the width of the dip. Here, it is used synonymously with the full width at half maximum (FWHM) of the dips. $C_{NV_{i}}$ are the contrast values of the individual dips.

The resonant frequency of a dip depends on the parallel component of an external magnetic field with respect to the corresponding NV-axis. An arbitrary external magnetic field $B_{a}$ therefore shifts the frequency of the dip by the vectorial component parallel to the NV-axis $B_{\left|\right|}$. To further characterize the output signal, one resonance is considered separately and Equation (3) is written as

(4) $$V_{T}\left(t\right)=V_{0}-\frac{C_{NV}}{1+\frac{4{\left(f_{MW}-\gamma_{e}B_{\left|\right|}\left(t\right)\right)}^{2}}{\Delta \nu^{2}}}$$

where $\gamma_{e}=h/\left(g_{e}\mu_{B}\right)$ is the gyromagnetic ratio with *h* as Planck’s constant, $g_{e}$ as the electronic g-factor and $\mu_{B}$ as the Bohr magneton.

The microwave frequency $f_{MW}$ can either be swept linearly, kept constant or be amplitude- or frequency-modulated (FM). Besides noise reduction, the use of FM has the advantage that the output signal is the derivative of the resonance spectrum after demodulation of the lock-in technique (cf. Figure 1c). The center frequencies of the resonances can be detected as zero crossings. Here, FM is used and is given by the carrier frequency $f_{c}$, the frequency of the local oscillator $f_{LF}$ and frequency deviation $f_{devi}$. $f_{LF}$ is used as the demodulation frequency $f_{ref}$ of the LIA. The FM modulated microwave as function of *t* is written as

(5) $$f_{MW}\left(t\right)=f_{c}+f_{devi}\;\cdot \sin\left(2\pi f_{LF}t\right)$$

$V_{T}$ can therefore be written as

(6) $$V_{T}\left(t\right)=V_{0}-\frac{C_{NV}}{1+\frac{4{\left(f_{c}+f_{devi}\sin\left(2\pi f_{LF}t\right)-\gamma_{e}B_{\left|\right|}\left(t\right)\right)}^{2}}{\Delta \nu^{2}}}.$$

With this equation, the frequency spectrum of specific points of the resonance spectrum can be modeled.

To simulate a realistic working point $B_{a}$ is set to a static component with a one-sided sinusodial offset $B_{a}\left(t\right)=B_{dc}+{\hat{B}}_{ac}/2\;\left(1+\sin\left(2\pi f_{B_{ac}}t\right)\right)$. With the projected magnetic field components to the NV-axis, the output signal is written as

(7) $$V_{T}\left(t\right)=V_{0}-\frac{C_{NV}}{1+\frac{4(f_{c}+f_{devi}\sin\left(2\pi f_{LF}t\right)-\gamma_{e}B_{dc||}-\gamma_{e}{\hat{B}}_{ac||}/2{\left(1+\sin\left(2\pi f_{B_{ac}}t\right)\right)}^{2}}{\Delta \nu^{2}}}$$

**Figure 6:**
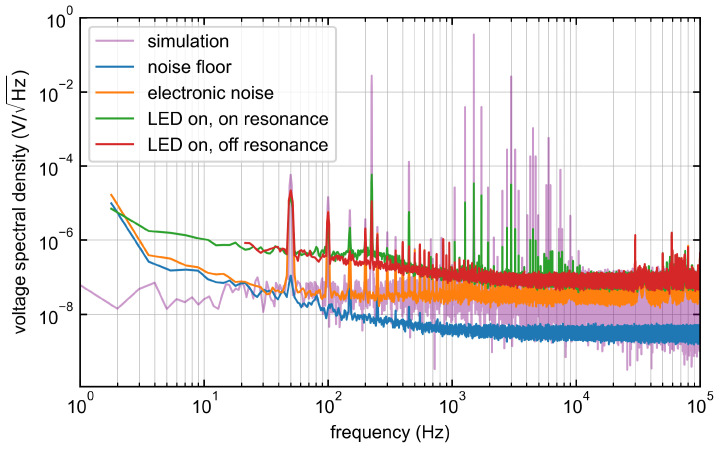
Measured voltage spectral density for different cases. Purple spectra: A simulated spectral density according to Equation (7) is shown whereas the amplitude is chosen arbitrarily. Blue spectra: the noise floor measured without any signal cable attached. Orange spectra: all devices running. LED is turned off. Red spectra: The LED is then turned on and the microwave is kept in a non-sensitive regime of 2.4GHz. Green spectra: the carrier frequency $f_{c}$ of the microwave is tuned to resonance. The frequency peaks follow the simulated frequency comb (purple).

A linear spectral density spectrum of the simulated time series is shown in Figure 6. Additional 50Hz harmonics (due to the European grid frequency) and a Gaussian noise were added to visualize the signal more realistically.

### 2.6. Measuring of Small Magnetic Field Changes

If $V_{T}$ is an FM-modulated signal and fed into an LIA, the demodulated output signal of the LIA $V_{LIA}$ is an approximation to the derivative of the input signal (cf. Figure 1c). In the immediate vicinity of the resonance $f_{res}$ and therefore the zero crossing of $V_{LIA}$, the demodulated signal of one resonance can be fitted linearly with the slope $m_{fit}$. In this range, the magnetic field parallel to one NV-axis is related to the signal output with:

(8) $$\Delta B_{\left|\right|}=\frac{\Delta V_{LIA}}{\gamma_{e}\cdot m_{fit}}.$$

$V_{LIA}$ is the amplitude output voltage multiplied by the sign of the phase Θ of the LIA and $m_{fit}$ the slope fitted to the demodulated spectrum. If the orientation of the diamond is not known, the applied magnetic field cannot be calculated directly by measuring only $\Delta B_{\left|\right|}$. In the measurement setup shown in Figure 1c. $B_{a}$ is limited to the known direction of the z-axis related to a coordinate system given by the direction of the Helmholtz coil. The NV-axis that is mostly parallel to the applied external field can easily be recognized by the largest Zeeman splitting. With a correction factor cos(θ) that can be determined by vector projection between $B_{\left|\right|}$ and one known magnetic field value of $B_{z}$, it is then possible to measure $\Delta B_{z}$ with only one resonance frequency

(9) $$\Delta B_{z}=\Delta B_{\left|\right|}/\cos\left(\theta \right)=\frac{\Delta V_{LIA}}{\gamma_{e}\cdot m_{fit}\cdot \cos\left(\theta \right)}\;\;.$$

## 3. Results

### 3.1. Crosstalk between MW-PCB and PD-PCB

With a vector network analyzer (ZNB8, Rhode & Schwarz, Munich, Germany) the transmission factor between the MW connector (port $S_{1}$) and the photodiode connector (port $S_{2}$) is measured. The frequency range is set from 2.4GHz to 3.4GHz. The $S_{12}$ parameter shows a high isolation value of $S_{12}=-51\;\mathrm{dB}$ over the entire used frequency range which proves analog shielding between MW and the photodiode signal.

### 3.2. Thermal Response

**Figure 4:**
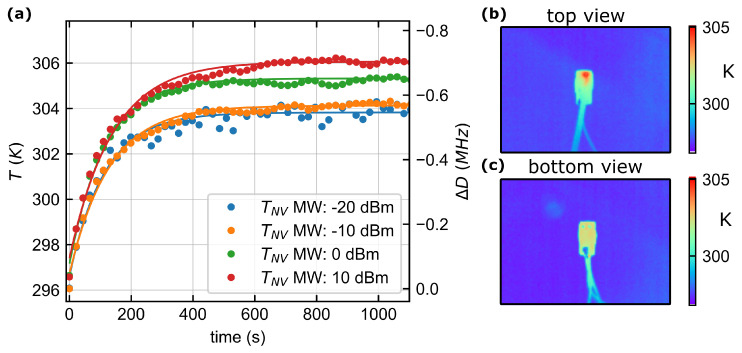
(**a**) Temperature drifts with different microwave power. Saturation is reached at temperature of the diamond $T_{NV}\approx 306\;K\left(32.85\;{}^{\circ}C\right)$ with 10dBm microwave power with reference to the ambient temperature of 296.2K(23.05°C). (**b**,**c**) Measurement of the surface temperature of the sensor at an ambient temperature of 296.9K(23.76°C) and with $P_{MW}=10\;\mathrm{dBm}$. Top view refers to the view of the LED-PCB and bottom view refers to the view of the PD-PCB. The largest temperature increase of $\Delta T_{sf}=8.1\;K$ is measured at a contact of the LED.

The temperature behavior of the sensor is studied. With an FM scan between 2.845GHz and 2.895GHz, the zero field splitting without an applied magnetic field is measured. The microwave power is varied between $P_{MW}=-20\;\mathrm{dBm}$ and $P_{MW}=10\;\mathrm{dBm}$. The shift of the ZFS, which is designated *D*, is measured. The shift is caused by the increased temperature [9] of the diamond due to the thermal and optical heat radiated by the LED and the heat induced by the microwave. The results are shown in Figure 4a. We observe a maximum shift of the ZFS of ΔD=−703kHz at $P_{MW}=10\;\mathrm{dBm}$ at a constant ambient temperature of 296.2K(23.05°C). Acosta et al. systematically investigated the behavior of the ZFS in the temperature range 280–330K [9] and found an average factor dD/dT=−74.2kHz/K for various diamonds with different concentrations. The diamond used is comparable to the diamonds used by Acosta due to its NV concentration. The factor is therefore used to determine the temperature shift within the diamond. We estimate a temperature increase of only $\Delta T_{NV_{max}}\approx 9.8\;K$ and a total temperature inside of the sensor of $T_{NV}\approx 306\;K\;\left(32.85\;{}^{\circ}C\right)$. The surface temperature of the sensor was measured with a thermal imaging camera (E40, Teledyne FLIR, Wilsonville, OR, USA) directed at both the LED-PCB (cf. Figure 4b) and the PD-PCB (cf. Figure 4c). The ambient temperature for these measurements is 296.9K(23.76°C). Outside the sensor, a maximum surface temperature of $T_{sf}=305\;K\;\left(31.85\;{}^{\circ}C\right)$ can be measured at a contact of the LED. $\Delta T_{sf}=8.1\;K$ is slightly lower than the measurements via the ZFS inside the sensor.

### 3.3. Shot Noise Limited Sensitivity (SNLS)

To further optimize the sensor performance, the parameters regarding the FM are varied. To compare different working points the shot noise limited sensitivity for a continuous wave (CW) application with a Lorentzian line shape [38,39] is calculated by

(10) $$\eta_{SNL}=\frac{4}{3\sqrt{3}}\frac{\gamma_{e}\Delta \nu }{C_{NV}\sqrt{R}}\;\;$$

where $4/3\sqrt{3}$ is a factor regarding the Lorentzian line shape, Δν is the full width half maximum (FWHM) of the resonance, $C_{NV}$ is the contrast of a resonance dip and *R* is the detected photon count rate. *R* is the photocurrent calculated by the output voltage of the TIA in the non-resonant case $V_{0}$ and the feedback resistor of the TIA [1]. To measure a resonance spectrum, an offset magnetic field of $B_{z}=6.1\;\mathrm{mT}$ is applied by an external Helmholtz coil. The resulting eight resonances were fitted with several Lorentz functions (Equation (3)) and the values for $C_{NV}$ and Δν were determined.

**Figure 5:**
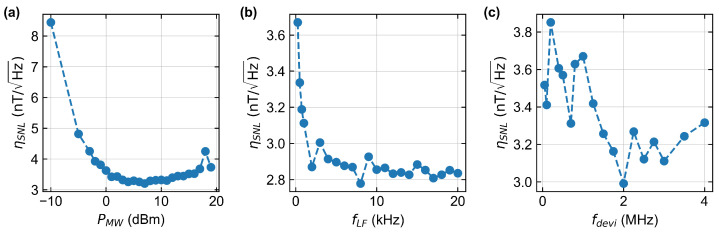
Shot noise limited sensitivity for different FM parameters. (**a**) Sweep of the microwave power from $P_{MW}=-10\;\mathrm{dBm}$ to $P_{MW}=19\;\mathrm{dBm}$. Almost consistently good results were achieved between $P_{MW}=4\;\mathrm{dBm}$ and $P_{MW}=11\;\mathrm{dBm}$. (**b**) The local oscillator frequency of the FM is swept between $f_{LF}=250\;\mathrm{Hz}$ and $f_{LF}=20\;\mathrm{kHz}$. The measured values correlate with the measured noise power density spectrum in Figure 6, which shows a similar decay and constant good values from leaving the 1/f noise at above approximately 1 kHz. (**c**) Sweep of the deviation used in the frequency modulation. $f_{devi}$ is changed between 50kHz and 4MHz. The best values are reached between $f_{devi}=1.75\;\mathrm{MHz}$ and $f_{devi}=3\;\mathrm{MHz}$.

First, the dependence of sensitivity with respect to microwave power $P_{MW}$ is investigated. Therefore the local oscillator frequency $f_{LF}$ is set to 1kHz and the frequency deviation $f_{devi}$ to 3MHz. The results show an increased contrast but also broadened linewidth of the resonances with increasing microwave power (cf. Figure 5a). Above $P_{MW}=10\;\mathrm{dBm}$, the sensitivity slowly decreases again.

The variation of the local oscillator frequency $f_{LF}$ is performed with $P_{MW}=10\;\mathrm{dBm}$ and $f_{devi}=2\;\mathrm{MHz}$ (cf. Figure 5b). When the 1/f noise is left the LF frequency has no measurable effect in the chosen frequency range. Δν and $C_{NV}$ remain nearly constant.

For the last analysis, the frequency deviation $f_{devi}$ is swept in the range of 50kHz and 4MHz (cf. Figure 5c). Since it was decided to work with microwave power that does not resolve the hyperfine structures of the diamond microcrystals used in CW applications, the most sensitive range differs from other publications [1,40]. The measurements show that to achieve $\eta_{SNL}\approx 3\;\mathrm{nT}$, the parameter set for the FM should be selected from the following: $P_{MW}=\left[5,10\right]\;\mathrm{dBm}$, $f_{devi}=\left[1.8,3\right]\;\mathrm{MHz}$ and $f_{LF}=\left[1.5,20\right]\;\mathrm{kHz}$.

### 3.4. Noise Measurement

In order to characterize the sensor further, the noise behavior of the sensor is investigated. The output voltage of the TIA is connected to the scope of the LIA without any external or internal filters and the bandwidth is set to 1MHz with a Hann window function. The voltage spectral density is measured and shown in Figure 6. The blue spectra are the noise floor without any signal cable. The orange spectra show the output voltage spectral density of the TIA with the connected sensor, the microwave generator running at 10dBm tuned to non-resonant case of about 2.4GHz and a magnetic field through the Helmholtz coil of $B_{a}\left(t\right)=6.11\;\mathrm{mT}+25\;\mu T\;\sin\left(2\pi \cdot 225\;\mathrm{Hz}\cdot t\right)$. 50Hz interference of the mains, as well as their harmonics, can be clearly seen here as in all spectra. The LED is then switched on and the microwave is operated off-resonant.

To show the noise behavior at a specific resonance, the carrier frequency $f_{c}$ of the FM microwave is set to resonance at $\gamma_{e}B_{dc||}$ following Equation (7). The spectrum is recorded again (green spectra). Figure 6 clearly shows the signal oscillating with the local oscillator frequency $f_{LF}=1.5\;\mathrm{kHZ}$ and the sinusoidal component of $B_{a}\left(t\right)$ with $f_{B_{ac}}=225\;\mathrm{Hz}$ signal added as side bands with harmonics which strongly correlate with the calculated purple spectra of Equation (7). Above 28kHz, interference caused by the TIA can be observed. In the current configuration, $f_{LF}$ is therefore limited to this frequency. According to Howard [41], uncorrelated power density spectra G(t,f) can be summed for t→∞ as $G_{Z_{\infty }}\left(f\right)=G_{X_{\infty }}\left(f\right)+G_{Y_{\infty }}\left(f\right)$. Therefore, the noise of the LED can be analyzed in more detail by looking at the difference between the spectral density $S_{EL_{\infty }}\left(f\right)=\sqrt{G_{EL_{\infty }}\left(f\right)}$ of the electronic without the LED and the spectral density $S_{I_{\infty }}\left(f\right)=\sqrt{G_{I_{\infty }}\left(f\right)}$ of the insensitive case where the LED is turned on. Leaving the 1/f regime the average spectral density of the LED in the frequency range $1\;\mathrm{kHz}<f^{*}<28\;\mathrm{kHz}$ is calculated as

(11) $$\left[{\bar{S}}_{LED}\left(f^{*}\right)\right]=\sqrt{{\left[{\bar{S}}_{I}\left(f^{*}\right)\right]}^{2}-{\left[{\bar{S}}_{EL}\left(f^{*}\right)\right]}^{2}}\;.$$

The result is that the spectral density of the LED is by factor 2.4 bigger then the spectral density of the remaining electronics and is therefore the dominant noise source in this case.

### 3.5. Magnetic Sensitivity

**Figure 7:**
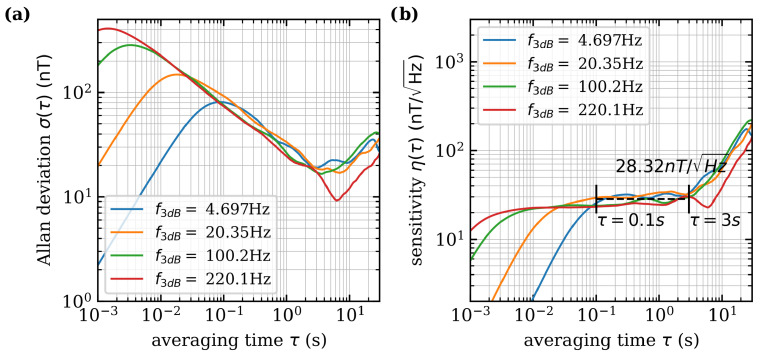
(**a**) The Allan deviation is measured for different cut-off frequencies of the LIA. The minimum detectable magnetic field change is the average minimum of all curves $\Delta B_{min}=15.44\;\mathrm{nT}$ (**b**) Sensitivity in relation to averaging time. Between τ=0.1s and τ=3s the mean sensitivity is $\eta =28.32\;\mathrm{nT}/\sqrt{\mathrm{Hz}}$.

The minimum detectable magnetic field and the sensitivity as a function of the integration time are determined using the Allan deviation. Allan deviation is a statistical metric commonly used to evaluate the deviation of measurement data grouped in intervals with an averaging time referred to as τ. Unlike standard deviation, Allan deviation systematically examines data sets with respect to the averaging time τ providing valuable insight into the noise characteristics of the system and the low-frequency drift behavior of the sensor. The Allan variance is calculated as [42]

(12) $$\sigma {\left(\tau \right)}^{2}=\frac{1}{2}\langle {\left({\bar{y}}_{n+1}-{\bar{y}}_{n}\right)}^{2}\rangle$$

where ${\bar{y}}_{n+1}$ and ${\bar{y}}_{n}$ are two adjacent mean values sampled over the sample time τ. The brackets 〈〉 denote an infinite time average. To reduce external influences such as magnetic noise induced by the Helmholtz coil, a permanent magnet is used to split the resonances. The microwave is tuned to resonance and demodulated data is recorded for 60s at a sample rate of 13.39k/s. The cut-off frequency of the LIA is varied between $f_{3dB}\approx 5\;\mathrm{Hz}$ and $f_{3dB}\approx 220\;\mathrm{Hz}$. Valid values only arise above the resulting time constant of the filter. The resulting Allan deviation is shown in Figure 7a and the sensitivity in relation to the integration time is shown in Figure 7b. The minimum detectable magnetic field change as an average of the minima of all four curves is $\Delta B_{min}=15.44\;\mathrm{nT}$. With short integration times τ, white noise dominates, which can be averaged out with longer integration times. The higher the cut-off frequency of the LIA is selected, the lower the absolute measurable field becomes. The mean sensitivity between τ=0.1s and τ=3s is $\eta =28.32\;\mathrm{nT}/\sqrt{\mathrm{Hz}}$. The increase in sensitivity above τ=2s shows that low-frequency components dominate at this point. This measurement shows a high sensitivity despite the unshielded environment.

### 3.6. Practical Application of Magnetic Field Measurement

To further determine the accuracy and precision of the sensor a known magnetic field is measured over time in two time series measurements. Two different magnetic fields are generated by the Helmholtz coil: (1) A square wave signal simulates switching processes. (2) A sinusoidal signal simulates a sinusoidal current.

For both measurements, an alternating component is added to the constant component of the current through the Helmholtz coil to generate a magnetic field $B_{z}=B_{z_{dc}}+\Delta B_{z}$. Since the orientation of the diamond to the magnetic field is not known, a correction factor must be assumed (Equation (9)) and is calculated for both measurements. A known magnetic field offset of $B_{z_{dc}}=6.11\;\mathrm{mT}$ is set, and $B_{\left|\right|}=5.94\;\mathrm{mT}$ is measured for the resonances with the largest shift (cf. Figure 1a). Therefore, the offset angle is calculated as θ≈13.6°.

For the first measurement, a square wave signal is added to the Helmholtz coil for the measurement time of ten seconds

(13) $$\Delta B_{z_{1}}\left(t\right)=6\;\mu T\cdot \frac{1+\mathrm{sign}(\sin(2\pi \cdot 0.5\;\mathrm{Hz}\cdot t))}{2}.$$

**Figure 8:**
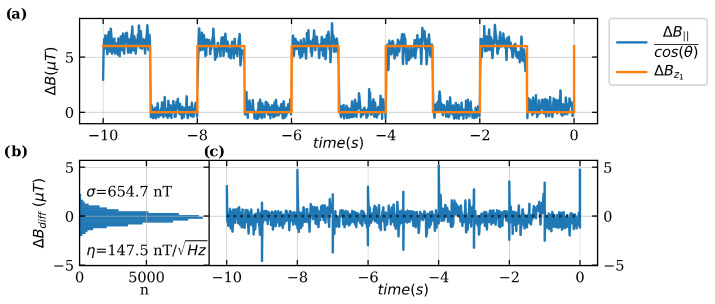
Measurement of a 0.5Hz square wave signal with $f_{LF}=3\;\mathrm{kHz}$, $f_{devi}=1\;\mathrm{MHz}$ and $P_{MW}=10\;\mathrm{dBm}$. $f_{c}$ is tuned to resonance. The LIA filter is set to 8th order and BWNEP=19.69Hz. The sample rate used is 6.67k/s. (**a**) Measured magnetic field component by the quantum sensor $\Delta B_{NV}$ corrected by the angle θ between NV-axis and direction of the applied magnetic field $B_{Z}$. (**b**) Histogram of the difference between $\Delta B_{Z}$ and $\Delta B_{NV}$. From the standard deviation σ=654.4nT and the BWNEP=19.69Hz the sensitivity is calculated as η=147.48nT. (**c**) Difference between $\Delta B_{Z}$ and $\Delta B_{NV}$ over time. Peaks at the switching edges result from the time constant of the filter.

Measurement and reference are shown in Figure 8a. The difference between $\Delta B_{z_{1}}$ and the corrected measurement signal is shown in Figure 8c. The histogram of the measured difference between reference and measurement signal is shown in Figure 8b. The standard deviation of σ=654.7nT and the resulting sensitivity is $\eta =\sigma /\sqrt{BWNEP}=147.5\;\mathrm{nT}/\sqrt{\mathrm{Hz}}$.

The second measurement is performed with a sinusoidal offset as

(14) $$\Delta B_{z_{2}}=30\;\mu T\cdot \left(1+\sin\left(2\pi \cdot 200\;\mathrm{Hz}\cdot t\right)\right)\;\;.$$

**Figure 9:**
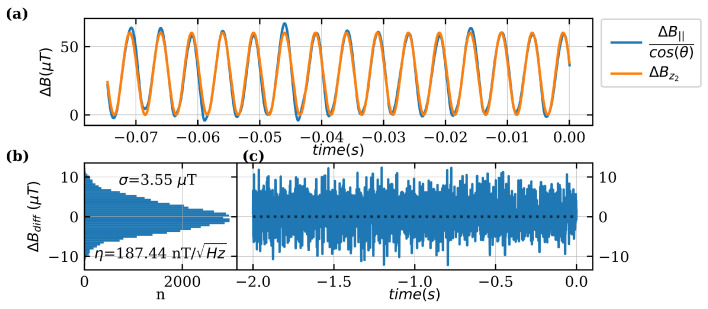
Measurement of a 200Hz sinusoidal signal. LIA sample rate and BWNEP were changed to 26.8k/s and BWNEP=359Hz. (**a**) Measured magnetic field component by the quantum sensor $\Delta B_{NV}$ corrected with the angle θ between NV-axis and direction of the applied magnetic field $B_{Z}$. (**b**) Histogram of the difference between $\Delta B_{Z}$ and $\Delta B_{NV}$. Resulting standard deviation σ=3.55μT and with BWNEP=359Hz the sensitivity is calculated as η=187.44nT. (**c**) Difference between $\Delta B_{Z}$ and $\Delta B_{NV}$ over time.

A reduced period of around 80ms of the whole measurement time of 2s is shown in Figure 9a as an example. Due to the higher bandwidth of the filter, the standard deviation is higher whereas the sensitivity with η=187.44nT is comparable to the measurement before. However, the measured sensitivity for both measurements differs from previous noise analyses of about factor 6. The main reason for that is visible deviations from the applied magnetic field. The Helmholtz coil is fed by a current source that provides a $\Delta I_{Coil}=1\;\mathrm{mA}$ at an offset of $I_{Coil}=932\;\mathrm{mA}$. The measured current value has a standard deviation of $\sigma_{current}=0.69\;\mathrm{mA}$ which results in a $\sigma_{B_{z}}=4.35\;\mu T$. In addition, all measurements were performed unshielded in a noisy environment.

## 4. Discussion

**Figure 10:**
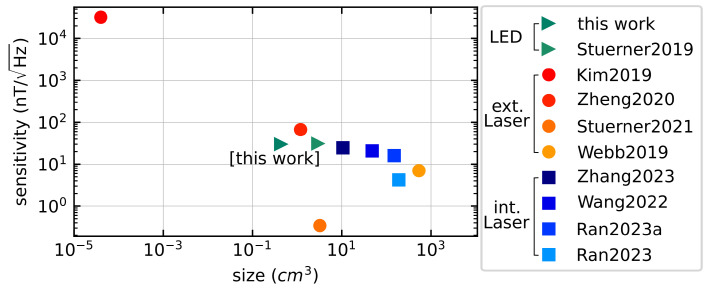
Selection of published sensors in terms of size and sensitivity. Only publications that have integrated a photodiode were considered. Also, publications using additional flux concentrators to improve sensitivity are not included. Furthermore, a distinction is made between fully integrated sensors (LED [32] or integrated laser [28,29,30,31]) and partial fiber-based sensors with external laser [1,24,26,27]. It can be seen that fully integrated LED-based sensors can currently be manufactured with smaller form factor.

In summary, we have developed a quantum sensor that is easy to build and performs similarly to previously published fully integrated and partly integrated sensors (cf. Figure 10). The use of commercially available components, the stacking of three commercially available PCBs, the use of a randomly orientated diamond microcrystal and the fact that no additional focusing optics are required shows that the sensor is easy to set up. Additionally, it simplifies the overall complexity and also the costs of the whole sensor system by providing an all-electric interface. Besides that, the sensor presented here is the smallest in the range of fully integrated sensors. The reached size of (6.9 × 3.9 × 15.9)$\;{\mathrm{mm}}^{3}$ reduces the overall volume by approx. factor 7 compared to the so far smallest fully integrated sensor by Stuerner et al. [32]. By using a diamond microcrystal, the magnetic field to be measured can be resolved finely by a volume of approx. $0.02\;{\mathrm{mm}}^{3}$. Comparing the cost of one 150μm high-pressure high-temperature (HPHT) diamond microcrystal with a $3\times 3\times 0.5\;{\mathrm{mm}}^{3}$ chemical vapor deposition (CVD) diamond (Thorlabs DNVB14) scaled to the same volume, it can be seen that diamond microcrystals are about 90% cheaper than CVD diamonds. The low internal heating by $\Delta T_{NV_{max}}\approx 9.8\;K$ in relation to the ambient temperature of 296.2K(23.05°C) is an advance in the field of integrated sensors based on NV centers. Due to the use of an LED, the power consumption is reduced to 3.3V·30mA=0.1W. The systematic study of the various FM parameters enabled the sensor system to be optimized to a shot noise limited sensitivity of $\eta_{SNL}=2.87\;\mathrm{nT}/\sqrt{\mathrm{Hz}}$. The achieved sensitivity of $\eta =28.32\;\mathrm{nT}/\sqrt{\mathrm{Hz}}$ opens up a wide range of possible applications; for example, as a current sensor in automotive engineering or switching cabinets with combined temperature monitoring. Possible applications are shown by two measurement sequences over time. The resulting sensitivities of $147.5\;\mathrm{nT}/\sqrt{\mathrm{Hz}}$ and $187.44\;\mathrm{nT}/\sqrt{\mathrm{Hz}}$ are in good relationship with the expected results. The standard deviation of the reference signal of $\sigma_{B_{z}}=4.35\;\mu T$ generated by the current source might be the main reason for the difference between the measurements previously described.

The noise measurement has also confirmed the calculation of the frequency spectrum of the TIA output signal (Equation (7)), which leads to a better understanding of the signal characteristics. This means that further signal processing can be better adapted to the expected signal. For example, $f_{LF}$ can be adjusted so that it matches the noise behavior of the TIA. Novel measurement sequences can also be developed that utilize the potential signal components contained in the harmonics of the frequency comb (Figure 6—purple curve).

In order to optimize the sensitivity, initial tests show that the pump light source can still be improved. The contrast $C_{NV}$ increases with increasing LED current. According to the LED manufacturer, a short-term current of up to 100mA is possible. It could be considered to operate the LED in pulsed mode to increase the contrast; however, influences on the diamond temperature must be taken into account. In addition, although a higher LED current has the effect that more pump light leads to higher fluorescence and thus also to a higher photocurrent, it also means that the proportion of pump light that is not filtered by the filter increases. This can lead to a deterioration in the SNR. At present, the positive effects outweigh the negative effects up to the 30mA range used. Furthermore, an effect on the LED can be observed at high MW powers above 15dBm. This indicates that the passive shielding above the LED should also be improved and could probably also lead to improvements in the lower power range. The calculation in Section 3.4 also shows that a reduction in the noise of the LED, e.g., through balanced photodetection, could further increase the sensitivity. Although the use of simple diamond microcrystals offers a great cost advantage, the use of less contaminated diamonds could reduce the line width and thus increase sensitivity. In the presented setup the LED is operated at its maximum rated continuous current, setting an upper bound for the emitted fluorescence. In a future designs, the use of a photodiode with a larger detector area could improve the detection efficiency and thereby increase the photocurrent. An increase in the photodiode current is favorable because an increase by a factor *n* leads to an increase in the signal-to-noise ratio (SNR) of $\sqrt{n}$, as the TIA is already operated in the shot noise limit.

In general, this work points out that quantum sensors based on NV centers have now really left the laboratory and can be widely used to contribute to applied research.

## Data Availability

Data underlying the results presented in this paper are not publicly available at this time but may be obtained from the authors upon reasonable request.

## Abbreviations

The following abbreviations are used in this manuscript:

NV	Nitrogen vacancy
ODMR	Optical detected magnetic resonance
LED	Light emitting diode
SMD	Surface mount device
HPHT	High pressure high temperature
CVD	chemical vapor deposition
MW	Microwave
PCB	Printed circuit board
FWHM	Full width half maximum
FM	Frequency modulation
LIA	Lock-in amplifier
CW	continuous wave
TIA	Transimpedance amplifier
CCS	Constant current source
SNLS	Shot noise limited sensitivity
